# Pomegranate Leaf Extract as a Source of Bioactive Compounds for Edible Coatings Applied to Strawberry Postharvest Preservation

**DOI:** 10.3390/polym18091100

**Published:** 2026-04-30

**Authors:** Daniela de Almeida Carrea, Farayde Matta Fakhouri, Fabricio Luiz Tulini, José Ignacio Velasco, Carmen Sílvia Favaro-Trindade

**Affiliations:** 1 College of Animal Science and Food Engineering (FZEA), University of Sao Paulo (USP), Pirassununga, São Paulo 13635-900, Brazil; danielacarrea@usp.br (D.d.A.C.); carmenft@usp.br (C.S.F.-T.); 2 Poly2 Group, Department of Materials Science and Engineering, Universitat Politècnica de Catalunya (UPC Barcelona Tech), 08222 Terrassa-Barcelona, Spain; jose.ignacio.velasco@upc.edu; 3 Centro das Ciências Biológicas e da Saúde (CCBS/UFOB), Universidade Federal do Oeste da Bahia, Barreiras 47810-047, Brazil

**Keywords:** starch, gelatin, residues, *Punica granatum* L., bioactive

## Abstract

This study investigated the use of aqueous pomegranate (*Punica granatum* L.) leaf extracts as a source of bioactive compounds in edible coatings for strawberry postharvest preservation. Extraction conditions were evaluated by varying solid-to-solvent ratio, temperature, and time, using total phenolic content (TPC) as the response variable. Response surface analysis indicated that the best predicted extraction conditions within the studied range were 1:50 (*w*/*v*), 57.36 °C, and 25 min. Among the evaluated treatments, extract C503 (1:50 (*w*/*v*), 50 °C, and 25 min) showed the highest experimental TPC (474.62 ± 21.69 mg GAE/g DM) and was selected for further characterization. This extract also showed high antioxidant capacity (FRAP: 7085 ± 72.0 µM FeSO_4_/g; ABTS: 4921 ± 149.0 µM Trolox/g) and antimicrobial activity against *Listeria monocytogenes* and *Staphylococcus aureus*. When incorporated into gelatin- and starch-based edible coatings and applied to strawberries, both coatings reduced mass loss and delayed deterioration during nine days of storage at room temperature. At the end of storage, mass loss was reduced by approximately 25% with the gelatin-based coating and 11% with the starch-based coating. These results support aqueous pomegranate leaf extract as a promising source of bioactive compounds for the development of sustainable edible coatings to improve strawberry preservation.

## 1. Introduction

Post-harvest fruit losses represent a major challenge for the global economy [1], mainly due to the short shelf life of fruits [2]. Fruit quality during storage is influenced by several factors, including temperature, packaging, microbial contamination, and handling conditions. Losses may result from respiration, transpiration, microbial deterioration, and physical damage occurring during production, transport, and commercialization. In particular, postharvest respiration, which is affected by temperature and by gases such as CO_2_, O_2_, and ethylene, accelerates senescence and contributes to quality deterioration [2,3].

The development of edible coatings [4,5,6] and films [7,8] has emerged as a sustainable alternative to conventional plastics with long degradation times. These materials, produced from natural or biodegradable polymers, may reduce the environmental impact associated with synthetic packaging while also helping to extend the shelf life of fruits and vegetables [6,9].

Starch is a polysaccharide and has been extensively studied in food packaging applications because of its abundance, low cost, renewability, and biodegradability [10,11,12]. In addition, starch-based matrices can incorporate natural compounds capable of improving both physical and functional properties. For instance, the addition of cranberry powder to arrowroot starch films altered the microstructure, reduced the glass transition temperature, and increased ascorbic acid content, while also providing natural color and flavor, features that may enhance the sensory appeal of packaging materials [8]. Similarly, the incorporation of spray-dried blackberry particles into starch films improved antioxidant activity, film flexibility, and solubility, highlighting the potential of starch-based materials for active packaging applications [7].

Starch-based materials have also proven effective in preserving fruit quality. Riva et al. (2020) [5] showed that the incorporation of lipids into starch-based coatings improved their moisture-barrier properties, making them particularly suitable for stone fruits. In addition, Fakhouri et al. (2015) [6] investigated corn starch-based biofilms combined with gelatin and demonstrated that these coatings effectively protected Red Crimson grapes during refrigerated storage by reducing weight loss and maintaining overall fruit quality.

Gelatin is another biopolymer widely used in edible films and coatings because of its film-forming ability and its compatibility with other biopolymers and additives [13,14,15]. Besides acting as a structural matrix, gelatin can improve film flexibility through its interaction with plasticizers, thereby reducing rigidity [16,17]. The incorporation of essential oils, such as *Melaleuca alternifolia*, into gelatin-based films has demonstrated strong antioxidant and antimicrobial activities, along with structural and functional improvements that contributed to extending the shelf life of refrigerated chicken breasts [18]. According to Fakhouri et al. (2003) [19], the addition of triacetin and fatty acids to gelatin-based films improves their barrier and mechanical properties without compromising transparency, particularly when used in composite structures.

Edible films and coatings may serve not only as structural barriers, but also as carriers of bioactive compounds such as plant extracts. The incorporation of these compounds can provide antioxidant and antimicrobial functionality, helping to delay oxidative and degradative processes, preserve product quality, and extend shelf life [9]. In this context, Bajaj et al. (2023) [4] showed that starch-based coatings containing plant extracts reduced weight loss and enhanced antioxidant properties, improving fruit preservation.

Pomegranate (*Punica granatum* L.) is rich in phenolic compounds and exhibits antioxidants, anti-inflammatory and antimicrobial activities. Extracts enriched in flavonoids and tannins have shown inhibitory effects against bacteria such as *Staphylococcus aureus* and *Escherichia coli* [20]. The use of pomegranate peels and leaves (which are usually considered inedible parts) as raw materials presents great potential in the food industry due to their low cost and the possibility of valorizing agricultural residues.

Previous studies indicate that pomegranate peel extracts are generally safe at moderate doses, showing no toxicity in rodents up to 600 mg/kg body weight [21]. Additionally, recent studies have shown that spray-dried hydroethanolic extracts from pomegranate leaves are safe, with no toxicity observed in animal models even at high doses up to 2000 mg/kg (acute) and 1000 mg/kg (subacute), while also exhibiting beneficial effects on lipid metabolism [22]. Therefore, replacing synthetic antioxidants with pomegranate peel or leaf extracts may represent a sustainable strategy for preserving food quality and extending shelf life [21].

In this context, the present study aimed to evaluate the effects of extraction conditions on the total phenolic content of aqueous pomegranate (*Punica granatum* L.) leaf extracts, to characterize the extract with the highest phenolic content in terms of antioxidant capacity and antimicrobial activity, and to incorporate this extract into gelatin- and starch-based edible coatings to evaluate their effect on extending the shelf life of strawberries.

## 2. Materials and Methods

### 2.1. Materials

The leaves of pomegranate were collected from pomegranate production at Magranatum in Valencia, Spain. The strawberries were purchased in the local market (Fres Tonic Producer, Rabat-Salé-Kénitra, Morocco) lot 1072599-4. Rice starch (Dayelet, Piera, Spain), gelatin 250 H 30, (Rousselot Gelatinas do Brasil, Amparo, Brazil), and Glycerol (C_3_H_8_O_3_).

### 2.2. Sample Preparation and Extraction

Pomegranate leaves were dried in an oven (LBX Instruments, Zaragoza, Spain) at 40 °C for 72 h. After drying, the leaves were manually ground in a mortar into small fragments, without further sieving, and stored in a dry, light-protected environment until extraction.

Aqueous extraction was carried out using distilled water as solvent at three solid-to-solvent ratios (1:10, 1:30, and 1:50, *w*/*v*), three temperatures (30, 50, and 70 °C), and three extraction times (15, 20, and 25 min) [20,23,24,25]. During extraction, a disposable tea filter (Finum Tea Filter, Hamburgo, Alemania) was used to retain the leaf powder while allowing solvent contact. Each extraction condition was independently prepared in triplicate. The resulting extracts were transferred to amber glass bottles, stored at 6 °C, and analyzed within 48 h.

### 2.3. Phenolic Compounds

The phenolic compounds in the extract were determined by means of the Folin–Ciocalteu method using spectrophotometry [26]; 0.25 mL of the sample extract was transferred to amber test tubes with 2.75 mL of a 3% Folin–Ciocalteu solution, and vortexed for 10 s. After resting for 5 min, 0.25 mL of a 10% sodium carbonate solution was added and vortexed. After resting for 60 min at room temperature (25 ± 2 °C), in triplicate, the absorbance was measured with a UV-visible spectrophotometer (ZUZI MODEL 4201/50, Auxilab, Beriáin, Spain) at 765 nm.

For spectrophotometer calibration, blank tubes were prepared with the extraction solvent, keeping the other experimental conditions. The results were calculated using a standard curve with gallic acid and expressed as gallic acid equivalents (AGE) per gram of dry mass (DM).

### 2.4. Antioxidant Evaluation

The antioxidant capacity of the aqueous extract was evaluated through FRAP (Ferric Reducing Antioxidant Power) and ABTS [2,2′-azino-bis(3-ethylbenzothiazoline-6-sulfonic acid)] assays.

For the FRAP analysis, the reagent solution was freshly prepared by mixing 25 mL of 0.3 M acetate buffer (pH 3.6), 2.5 mL of 10 mM TPTZ solution (dissolved in 40 mM HCl), and 2.5 mL of 20 mM ferric chloride solution. Aliquots of 90 µL of extract were added to test tubes containing 270 µL of distilled water and 2.7 mL of FRAP reagent. The mixture was vortexed and incubated in a water bath at 37 °C for 30 min. Absorbance was measured at 595 nm using a UV-Vis spectrophotometer (ZUZI MODEL 4201/50, Auxilab Beriáin, Spain), with the FRAP reagent serving as a blank. A standard curve was established using ferrous sulfate solutions, and results were expressed as µM of ferrous sulfate equivalents per gram of sample [27].

In the ABTS assay, the radical cation solution was prepared by reacting 5 mL of 7 mM ABTS stock solution with 88 µL of 140 mM potassium persulfate, followed by incubation in the dark for 16 h. The resulting solution was diluted with ethanol to an absorbance of 0.7 ± 0.02 at 734 nm. Then, 30 µL of the sample was added to 3 mL of the ABTS solution, vortexed, and left to react in the dark for 6 min. Absorbance was recorded at 734 nm, with ethanol as the blank. A calibration curve was generated using Trolox standards at concentrations ranging from 100 to 2000 µM. The antioxidant capacity was expressed as µM Trolox equivalents (TE) per gram of sample [28].

### 2.5. Antimicrobial Activity

For this antimicrobial assay, the following strains were used: *Enterococcus faecalis* ATCC 29212, *Escherichia coli* ATCC 29212, *Klebsiella pneumoniae* ATCC 700603, *Listeria monocytogenes* NCTC 13627 DSM 19094, *Pseudomonas aeruginosa* ATCC 27853, *Salmonella enterica subsp. enterica serotype Enteritidis* NCTC 6676, *Staphylococcus aureus* NCTC 12493 WDCM 00212, *Candida albicans* ATCC 10231, *Aspergillus brasiliensis* ATCC16404, *Botrytis cinerea* FAT1252, *Penicillium roqueforti* ATCC 10110 and *Trichoderma reesei* ATCC 26921. The antimicrobial activity was evaluated using the disk diffusion method based on the CLSI methods M02, M44 and M51, with modifications [29,30,31]. A volume of 10 µL of extracts (100 mg/mL in 5% DMSO) was applied to 5 mm sterile disks, which were then placed on Mueller–Hinton agar previously inoculated with 100 µL of a microorganism suspension adjusted to an absorbance of 0.1. The plates were incubated at 35 °C for 24 h for bacterial and yeast strains, or at 28 °C for 96 h for mold strains. The assay was conducted in triplicate using a 5% (*v*/*v*) DMSO aqueous solution as negative control, and the diameter of the inhibition halos was measured in millimeters.

### 2.6. Preparation of Edible Coatings

Two types of coating were prepared using the pomegranate leaf extract corresponding to treatment C503, which showed the highest experimental total phenolic content among the evaluated conditions. For the starch-based film-forming solution, 3 g of rice starch was mixed with 20% glycerin (based on dry starch weight) and 100 mL of pomegranate extract, followed by heating at 70 °C for 20 min until starch gelatinization. For the gelatin-based film-forming solution, 5 g of gelatin and 10% glycerin were added to 100 mL of extract; the mixture was allowed to stand for 1 h and then heated in a water bath at 70 °C for 5 min.

### 2.7. Application on Strawberries

Strawberries were coated using the immersion technique. Fruits from the same batch were separated and submerged in the film-forming solution at room temperature for 1 min, then dried at the same temperature. Uncoated strawberries from the same lot were washed with distilled water and used as the control. In this study, fruits were stored at room temperature (25 °C, 50% relative humidity) for 9 days to better simulate realistic postharvest conditions encountered during distribution and at the point of sale, such as in markets.

#### 2.7.1. Strawberry Mass Loss

Five samples of each coating and control were placed on identified plates and weighed daily on an analytical scale (Pioneer, Ohaus Corporation, Parsippany, NJ, USA). The mass loss was calculated using Equation (1).

(1) $$Mass\;Loss\;(\%)=\frac{{m_{i}-m}_{f}}{m_{i}}\times 100$$

where $m_{i}$ is the initial fruit mass at the beginning of storage and $m_{f}$ is the fruit mass measured on the evaluation day.

#### 2.7.2. Brix

The Brix degree was measured using a refractometer (iitrust Brix 0–90%, C03270, China), the strawberries were placed in cotton, and the liquid was placed in the refractometer for measurement.

#### 2.7.3. Moisture

Strawberries coated with each type of coating, as well as the control samples, were tested daily. Three grams (3 g) of each sample, in triplicate, were placed in a convective oven at 105 °C for 24 h. After drying, the samples were placed in a desiccator containing silica gel until cooled, and then weighed. On the last day, five strawberries from the mass loss experiment were also tested. Equation (2) was used to calculate moisture content, and Equation (3) was used to calculate total solids.

(2) $$Moisture\;(\%)=\frac{{m_{1}-m}_{2}}{m_{1}}\times 100$$

where $m_{1}$ is the initial sample mass before drying and $m_{2}$ is the sample mass after drying.

Total Solids (%) = 100 − Moisture (%)(3)

#### 2.7.4. pH

The pH was evaluated in samples daily, measured using a pH meter (M21 Metric, Allela, Spain) according to the methodology described by AOAC (2005) [32].

### 2.8. Statistical Analysis

The response variable was total phenolic content (TPC), expressed as mg gallic acid equivalents per gram of dry matter (mg GAE/g DM). The response surface methodology was applied using a full 3^3^ factorial design to evaluate the effects of concentration, temperature, and extraction time on TPC. The independent variables were coded at three levels (−1, 0, and +1), corresponding to concentration (1:10, 1:30, and 1:50, *w*/*v*), temperature (30, 50, and 70 °C), and time (15, 20, and 25 min), respectively. A second-order polynomial model including linear, quadratic, and interaction terms was fitted to the data. Model adequacy was assessed by analysis of variance, coefficient of determination (R^2^), adjusted R^2^, residual analysis, and lack-of-fit testing. Response surface and contour plots were generated to visualize the effects of the factors, and numerical optimization was used to estimate the experimental condition associated with maximum predicted TPC.

The results obtained from the coatings were submitted to analysis of variance (ANOVA) and Tukey’s test using R (RStudio (version 4.6.0)) at 5% significance.

## 3. Results and Discussion

### 3.1. Characterization of the Extract

**Figure 1:**
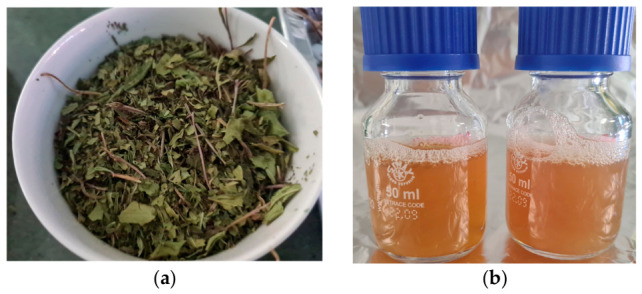
(**a**) Pomegranate dry leaves and (**b**) aqueous extract.

The mild drying conditions preserved the dark coloration of the leaves, which remained similar to that of the fresh, non-dried leaves (Figure 1). The resulting aqueous extract, however, exhibited a yellowish color.

#### 3.1.1. Optimization of Extraction Conditions

The extraction conditions were evaluated using response surface methodology (RSM) to determine the influence of solvent concentration, temperature, and extraction time on total phenolic content (TPC). A second-order polynomial model was fitted to the experimental data, allowing TPC to be described as a function of the coded variables.

The analysis of variance (ANOVA) results are presented in Table 1. The model was statistically significant (*p* < 0.001), confirming that the selected quadratic model adequately describes the experimental system. The linear terms exhibited a strong contribution (F = 44.605; *p* = 2.85 × 10^−8^), indicating that the main effects of the independent variables govern the extraction process. In addition, the quadratic terms were also significant (F = 8.790; *p* = 9.65 × 10^−4^), demonstrating the presence of nonlinear behavior and suggesting the existence of optimal extraction conditions within the studied range. Conversely, the interaction terms were not significant (*p* > 0.05), indicating that the combined effects of the variables are less pronounced compared to their individual contributions.

The model exhibited a coefficient of determination (R^2^ = 0.905) and an adjusted R^2^ of 0.855, indicating good predictive capability and agreement between experimental and predicted values. The pure error estimation, obtained from replicate experiments, yielded a mean square of 417.09, reflecting the intrinsic variability of the experimental system and supporting the reliability of the fitted model.

The regression coefficients are summarized in Table 2. Among the studied variables, solvent concentration (x_1_) showed the highest positive effect on TPC (*p* < 0.001), followed by extraction time (x_3_) and temperature (x_2_). This behavior suggests that increased solvent-to-solid ratio enhances the diffusion of phenolic compounds from the matrix, while longer extraction times promote mass transfer equilibrium. The significant quadratic effects of concentration (x_1_^2^) and temperature (x_2_^2^) indicate that excessive levels of these parameters may lead to reduced efficiency, possibly due to saturation effects or thermal degradation of phenolic compounds.

Based on the regression coefficients shown in Table 2, the fitted second-order polynomial model describing TPC as a function of the coded variables can be expressed as:

TPC = 183.2609 + 106.5600 x_1_ + 36.6057 x^2^ + 53.1580 x_3_ + 2.1617 x_1_ x_2_ + 15.1114 x_1_ x_3_ + 10.8497 x_2_ x_3_ + 65.3663 x_1_^2^ − 67.4231 x_2_^2^ + 18.8946 x_3_^2^

where x_1,_ x_2_, and x_3_ represent the coded variables corresponding to solvent concentration, temperature, and extraction time, respectively.

This equation can be used to predict TPC within the studied experimental domain and to identify optimal extraction conditions.

**Figure 2:**
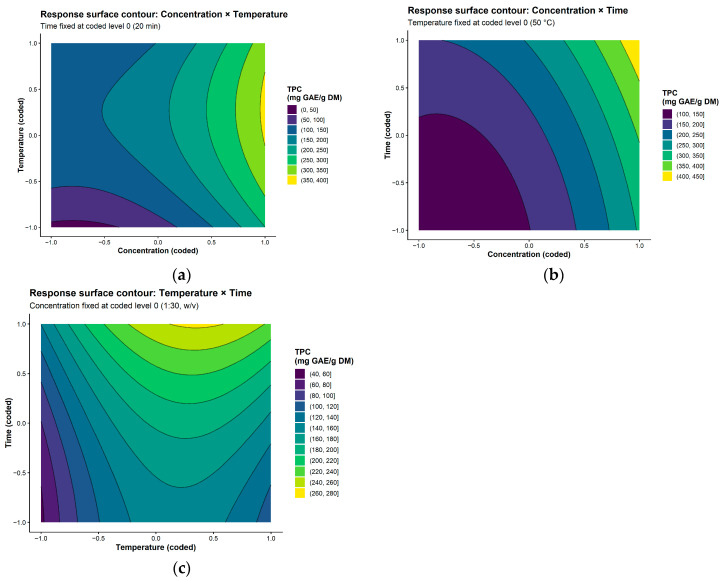
Contour plots showing the effects of (**a**) concentration and temperature, (**b**) concentration and time, and (**c**) temperature and time on total phenolic content (TPC). The third variable was kept constant at its central level.

**Figure 3:**
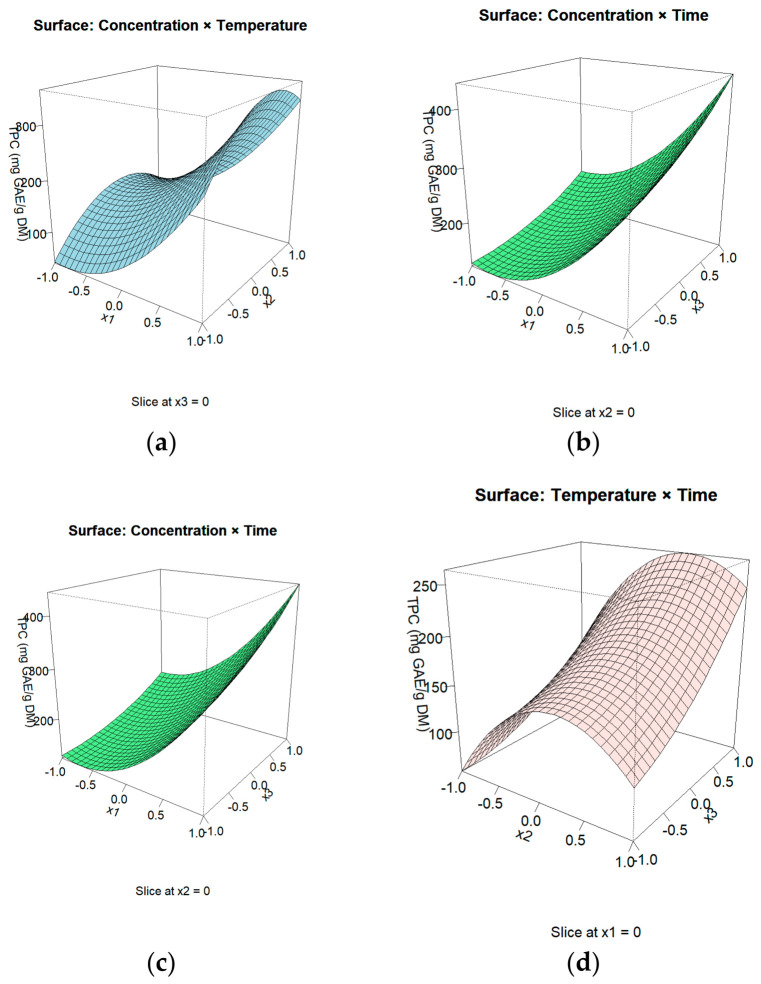
Three-dimensional response surface plots illustrating the combined effects of process variables on total phenolic content (TPC). (**a**) concentration and temperature, (**b**) concentration and time, (**c**) concentration and time and (**d**) temperature and time.

The response surface behavior is illustrated by the contour plots (Figure 2) and three-dimensional response surfaces (Figure 3). The contour plots indicate that maximum TPC values are achieved at high solvent concentration and longer extraction times, while temperature shows an optimal intermediate range. The curvature observed in the response surfaces is consistent with the significance of the quadratic terms. The absence of pronounced elliptical contour patterns confirms the limited contribution of interaction effects.

The three-dimensional plots (Figure 3) further highlight a region of maximum response at high solvent concentration and extended extraction time under moderate thermal conditions. The model predicted the highest TPC at a solvent concentration of 1:50 (*w*/*v*), temperature of 57.36 °C, and extraction time of 25 min, with a predicted value of 451.48 mg GAE/g DM. Because this predicted maximum was located near the upper boundary of the experimental domain, these conditions should be interpreted as the best predicted extraction conditions within the studied range.

Overall, the RSM approach was useful for describing the influence of the extraction variables on TPC and for identifying the combination of conditions associated with greater phenolic recovery. Consistent with the model prediction, Tukey’s test showed that C503 differed significantly from all other treatments (*p* < 0.05), confirming that this treatment yielded the highest experimental TPC among the evaluated conditions (Table 3).

The total phenolic content in the pomegranate leaf extracts obtained in this study ranged from 42.73 ± 3.95 to 474.62 ± 21.69 mg GAE/g of DM, values higher than those reported in several studies in the literature. These results were achieved using aqueous extracts, which are safer and more suitable for food applications compared to organic solvents. Balamurugan et al. (2020) [33] reported 220.83 mg GAE/g using methanol, while Sharayei et al. (2019) [34] found values between 10.69 and 69.55 mg GAE/g with various extraction solvents. Similarly, Morzelle (2012) [35] obtained 61.8 mg GAE/g in aqueous extracts. Studies involving different pomegranate cultivars Garima et al. (2009) [36], reported an average of 359 mg GAE/g DM. By contrast, Nag et al. (2018) [37] found much lower values, ranging from 5.36 to 18.34 mg GAE/g of solids.

The results obtained in the present study were also consistent with those reported by Trabelsi et al. (2020) [20], who observed values ranging from 122 to 382 mg GAE/g dry mass in different pomegranate leaf extracts, with the aqueous extract reaching 262.98 mg GAE/g DM. Together, these findings reinforce that extraction parameters, particularly temperature, time, and concentration, play a decisive role in the recovery of bioactive compounds from pomegranate leaves.

#### 3.1.2. Antioxidant Analysis

The antioxidant activity results for the aqueous pomegranate leaf extract obtained under the best-performing extraction condition are presented in Table 4.

For the ABTS assay, the values obtained for the aqueous pomegranate leaf extract in the present study were higher than those reported by Yu et al. (2021) [38] for pomegranate leaf infusions, which ranged from 1660 to 1800 (μmol Trolox g^−1^ DW). The ABTS values observed here were also higher than those reported by Pengkumsri et al. (2019) [39] for an aqueous pomegranate peel extract (1311.55 ± 25.53 mg TEAC/g sample). In contrast, Morzelle (2012) [35] reported higher ABTS values for different pomegranate extracts, ranging from 9221.76 to 9779.39 (μM Trolox/g extract). These comparisons indicate that antioxidant capacity may vary considerably according to the pomegranate fraction analyzed and the extraction system employed. In this regard, Pengkumsri et al. (2019) [39] showed that extraction method strongly influences phytochemical content and antioxidant activity, with the highest antioxidant activity being observed in acidified ethanolic extracts of pomegranate peel. Likewise, Morzelle (2012) [35] discussed that extraction yield and antioxidant response depend on solvent polarity and solubility, and that no single solvent system is suitable for recovering all bioactive compounds from a plant matrix. In agreement with this, Yu et al. (2021) [38] demonstrated that aqueous pomegranate leaf infusions exhibit strong antioxidant activity and good stability over storage, which the authors associated with transformations in the polyphenolic profile and compensation of antioxidant activity among compounds. In addition, extract C703 showed a lower ABTS value than C503, with 1946.61 (μM Trolox/g), further supporting the influence of extraction conditions on antioxidant response.

For the FRAP assay, Yu et al. (2021) [38] found values between 2030 and 2230 (μmol Trolox g^−1^ DW) for pomegranate leaf infusions, which were lower than the value obtained in the present study for the aqueous pomegranate leaf extract. Silva et al. (2023) [40] also reported a high FRAP value for pomegranate leaf hydroethanolic extracts (6448.44 ± 524.05 μmol FeSO_4_ equivalents g^−1^ DW), exceeding the values observed for peel and seed fractions in the same study. Although the extraction system was hydroethanolic rather than strictly aqueous, this finding further supports the strong antioxidant potential of pomegranate leaves. Sharayei et al. (2019) [34] reported values ranging from 287 to 1951 (μM Fe^2+^/g) in different pomegranate peel extracts, with 1432 (μM Fe^2+^/g) for the aqueous extract, while Nascimento et al. (2013) [41] also reported high antioxidant capacity for pomegranate extracts, with FRAP values of 8566.67 (μM ferrous sulfate/mL) for the aqueous extract. Taken together, these studies reinforce that antioxidant response in pomegranate-derived extracts is markedly dependent on the plant material and extraction conditions.

#### 3.1.3. Antimicrobial Activity of the Selected Aqueous Extract

**Figure 4:**
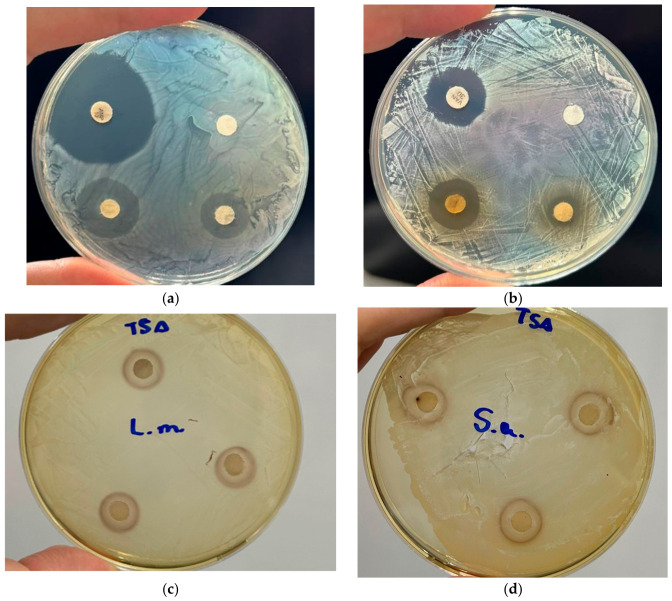
Disk diffusion assay showing (**a**) the control plate for *Listeria monocytogenes*, including the positive control (ampicillin, 10 µg), negative control (water), and extract disks at 20 and 10 µL; (**b**) the control plate for *Staphylococcus aureus*, including the positive control (vancomycin, 30 µg), negative control (water), and extract disks at 20 and 10 µL; (**c**) the inhibition zone produced by selected aqueous extract (C503) extract against *Listeria monocytogenes*; and (**d**) the inhibition zone produced by selected aqueous extract (C503) extract against *Staphylococcus aureus*.

Inhibitory activity was detected only for *L. monocytogenes* (17.3 ± 0.5 mm) (Figure 4c) and *S. aureus* (11.5 ± 0.5 mm) (Figure 4d). No inhibitory halo was observed for the negative control or for the other strains evaluated in this study.

Previous studies have shown that *Punica granatum* L. leaf extracts obtained using different extraction systems may exhibit antimicrobial activity against both Gram-positive and Gram-negative bacteria, although the magnitude of this effect varies according to extract type and bacterial strain [42]. Trabelsi et al. (2020) [20] reported that pomegranate leaf extracts, including the aqueous extract, inhibited S. aureus and E. coli, but with variable efficacy depending on the strain, with lower activity of the aqueous extract against resistant bacteria. In the same study, the extract enriched in total oligomer flavonoids showed the strongest antimicrobial effect. Likewise, Singh et al. (2023) [21] reported antibacterial activity for methanolic Punica granatum L. leaf extracts against multiple bacterial strains. Taken together, these results indicate that the antimicrobial response of pomegranate leaf extracts is influenced by the extraction procedure, extract composition, and susceptibility of the target microorganism, which may explain the selective activity observed for the aqueous extract in the present study.

### 3.2. Strawberry Coatings

**Figure 5:**
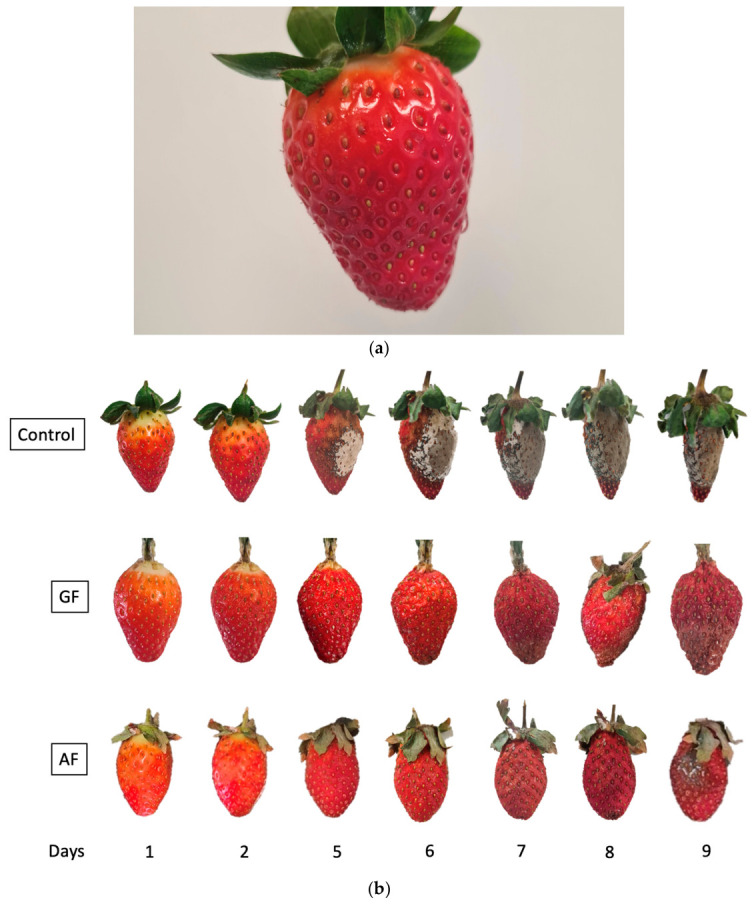
(**a**) Coated strawberry; (**b**) strawberries coated during storage. GF: Gelatin-based coating; AF: starch-based coating.

After the application, all the strawberries appeared visually similar, with the use of pomegranate extract in the edible coatings not affecting the color of the strawberries (visually). The coatings were homogeneous, transparent and unnoticeable (Figure 5a). During storage, visible changes in relation to fungal growth on the samples were observed starting on day four. The control strawberries began to show fungal growth, while in the samples with starch-based coatings, fungi became visible on day five on the back of the strawberries (Figure 5b). However, for the samples covered with gelatin and pomegranate aqueous extract, no fungal growth was observed throughout the 9 days of storage.

These results suggest that the coating acted as a physical barrier, reducing oxygen exposure and, consequently, slowing down the respiration rate and senescence of the strawberries. By limiting oxygen availability, the coating also hindered fungal development, as most fungi require oxygen to grow. As a result, the gelatin-based coating containing pomegranate leaf extract effectively delayed fruit ripening and microbial spoilage, extending the shelf life of strawberries by 3 days. According to Zebua et al. (2025) [43], strawberries treated with the coating maintained higher ascorbic acid levels compared to the control, particularly by day 4 of storage. The authors emphasized the role of antioxidant-rich coatings in slowing vitamin C degradation, supporting the potential of gelatin-based films as a protective barrier against oxidative losses in fruits.

#### 3.2.1. pH, Moisture and Total Solids Content of Strawberries over Time

The pH remained relatively stable over the 9 days of storage, ranging from 2.93 to 3.51, with no significant differences among treatments (*p* > 0.05). These values are consistent with those reported in previous studies on coated strawberries, which generally range from 3.0 to 4.0 [44,45,46]. The absence of significant pH changes indicates that the coatings did not adversely affect the natural acidity of the fruit during storage.

In addition to pH, soluble solids content was monitored as an indicator of fruit maturation and dehydration. Overall, °Brix values increased during storage in all treatments (Table 5), which may be associated with the concentration of soluble sugars as ripening progressed and moisture content decreased. On day 8 and 9, °Brix could not be determined for some samples because the fruits were excessively deteriorated and dry. Similar behavior has been reported in previous studies, in which °Brix values increased over time across treatments, ranging from 5.4 to 19.4 °Brix [44,45].

Regarding moisture, the coated strawberries (GF and AF) did not differ significantly from the control on the first day of storage (*p* > 0.05). Moisture decreased in all treatments during storage, with the greatest decline observed in the control group. At the end of storage, the control showed a moisture content of 51.63%, whereas GF and AF exhibited higher values of 82.19% and 78.63%, respectively. Higher moisture levels are important because they are associated with the preservation of fruit texture and fresh appearance [44].

**Figure 6:**
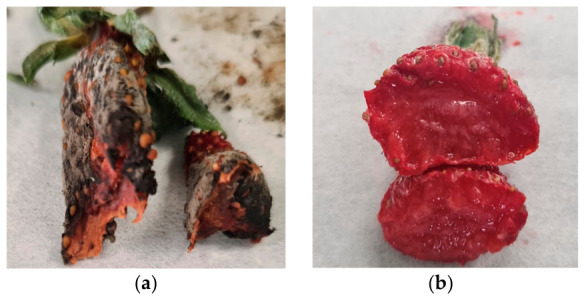
Cross-sectional appearance of strawberries on day 9 of storage: (**a**) control and (**b**) gelatin-based coating.

The moisture values observed in this study are consistent with those reported in the literature. Françoso et al. (2008) [44] reported a moisture content of 93% in irradiated strawberries, a value comparable to that recorded on the first day of storage in the present study. Likewise, da Silva and Schmidt (2015) [45] showed that edible coatings do not necessarily result in greater moisture retention. In their study, moisture decreased in all treatments during storage; however, the control maintained higher moisture values (93.19–86.97%) than strawberries coated with cassava starch acetate (93.45–80.21%) and cassava starch acetate plus potassium sorbate (93.25–76.64%). These findings indicate that the effect of edible coatings on moisture retention depends on formulation and storage conditions. Under the room-temperature conditions of the present study, both GF and AF showed higher final moisture content than the control (Figure 6).

The reduction in moisture content during storage was accompanied by changes in soluble solids, as reflected by the °Brix values. Under non-refrigerated conditions (25 °C), these parameters varied together during storage. Therefore, moisture content, °Brix, and mass loss should be interpreted as related parameters of strawberry postharvest behavior, although they should not be considered equivalent measurements.

#### 3.2.2. Mass Loss of Strawberries During Storage

**Figure 7:**
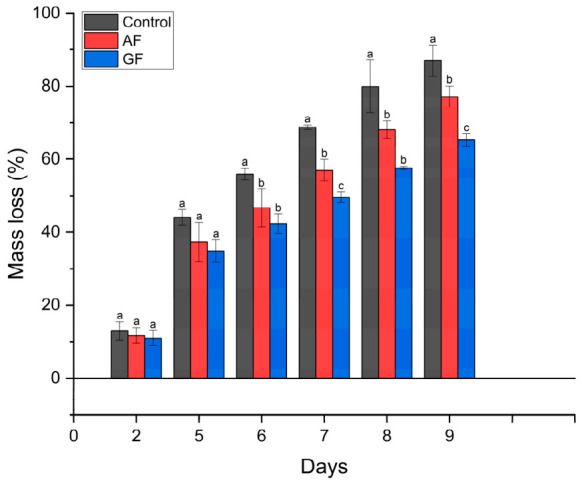
Mass loss of control and coated strawberries. AF: Starch-based coating; GF: gelatin-based coating. Error bars represent standard deviation (*n* = 3). Different letters indicate significant differences among treatments on the same storage day according to Tukey’s test at the 5% significance level.

Figure 7 shows the percentage of mass loss during storage for the control, AF and GF treatments. Significant differences among treatments were observed, with both coatings showing lower mass loss than the control. By day 6, AF and GF had already reduced mass loss relative to the control by approximately 17% and 24%, respectively, suggesting a lower rate of water loss during the early stages of storage.

By day 9, the control showed the highest mass loss (87%), whereas strawberries coated with AF and GF reached approximately 77% and 65%, respectively. Overall, both coatings reduced mass loss during storage, with GF showing the strongest effect over the evaluated period, corresponding to an approximately 25% reduction relative to the control. Although AF was less effective than GF, it still reduced mass loss by approximately 11% compared with the control, indicating a partial barrier effect that contributed to delaying dehydration during storage.

Previous studies have shown that gelatin-based coatings alone can slightly reduce weight loss in strawberries. However, the incorporation of additional compounds is often required to obtain a more pronounced preservation effect. For example, Aitboulahsen et al. (2018) [47] reported that, although a gelatin-based coating provided limited protection, the addition of peppermint essential oil markedly enhanced its performance, reducing weight loss from 24% in the control to only 3% after 13 days of storage. In contrast, Korte and Favarão (2016) [46] observed that gelatin-based coatings containing peppermint or clove extracts did not reduce mass loss and, in some cases, resulted in higher losses than the control. Taken together, these studies indicate that the effectiveness of gelatin-based coatings in limiting mass loss depends strongly on formulation and storage conditions.

Although the present study focused on the application of coatings directly on fruit, where performance depends on the interaction between the coating and the fruit surface, the characterization of standalone films (e.g., mechanical, barrier, and solubility properties) would provide valuable complementary insights into the structure–property relationships governing coating functionality.

## 4. Conclusions

The extraction conditions markedly affected the recovery of phenolic compounds from aqueous pomegranate (*Punica granatum* L.) leaf extracts. Response surface analysis indicated that the best predicted extraction conditions within the studied range were 1:50 (*w*/*v*), 57.36 °C, and 25 min. Among the evaluated treatments, C503, corresponding to 1:50 (*w*/*v*), 50 °C, and 25 min, showed the highest experimental total phenolic content and was therefore selected for further characterization. The selected extract also exhibited high antioxidant capacity, consistent with its elevated phenolic content, and selective antimicrobial activity against *Listeria monocytogenes* and *Staphylococcus aureus*, confirming the potential of pomegranate leaves as a source of bioactive compounds obtained using water as the extraction medium.

When incorporated into edible coatings, the extract contributed to improved strawberry preservation during storage. Both the gelatin- and starch-based coatings reduced mass loss relative to the control, with the gelatin-based coating showing the best overall performance. At the end of storage, mass loss was reduced by approximately 25% with the gelatin-based coating and 11% with the starch-based coating relative to the control. Although no inhibition of *Botrytis cinerea* was observed in the in vitro antimicrobial assay, the gelatin-based coating containing the extract reduced visible fungal growth on the fruit surface during storage.

Overall, the results support the potential of aqueous pomegranate leaf extract and biopolymer-based coatings as a sustainable strategy for strawberry preservation, while also highlighting the importance of extraction conditions and coating formulation in determining their effectiveness.

## Figures and Tables

**Table 1:** Analysis of variance (ANOVA) for the quadratic model fitted to total phenolic content (TPC).

Source	DF	Sum of Squares	Mean Square	F-Value	*p*-Value	Significance
Linear terms	3	279374	93125	44.605	2.85 × 10^−8^	***
Interaction terms	3	4209	1403	0.672	0.5809	ns
Quadratic terms	3	55054	18351	8.790	9.65 × 10^−4^	***
Residual error	17	35492	2088	—	—	—

*** *p* < 0.001; ns: not significant.

**Table 2:** Regression coefficients of the quadratic model for total phenolic content (TPC).

Term	Estimate	Std. Error	t-Value	*p*-Value	Significance
Intercept	183.261	23.265	7.877	4.50 × 10^−7^	***
x_1_ (Concentration)	106.560	10.770	9.894	1.81 × 10^−8^	***
x_2_ (Temperature)	36.606	10.770	3.399	3.42 × 10^−3^	**
x_3_ (Time)	53.158	10.770	4.936	1.25 × 10^−4^	***
x_1_x_2_	2.162	13.190	0.164	0.872	ns
x_1_x_3_	15.111	13.190	1.146	0.268	ns
x_2_x_3_	10.850	13.190	0.823	0.422	ns
x_1_^2^	65.366	18.654	3.504	2.72 × 10^−3^	**
x_2_^2^	−67.423	18.654	−3.614	2.14 × 10^−3^	**
x_3_^2^	18.895	18.654	1.013	0.325	ns

*** *p* < 0.001; ** *p* < 0.01; ns: not significant.

**Table 3:** Total phenolic compounds in pomegranate leaf extracts.

Extract Code *	Concentration (*w*/*v*)	Temperature (°C)	Time (min)	mg GAE/g DM
C301	1:50	30	15	175.66 ± 19.41 ^hij^
C302	1:50	30	20	234.22 ± 26.63 ^fgh^
C303	1:50	30	25	330.95 ± 19.69 ^cd^
C501	1:50	50	15	312.86 ± 27.81 ^cde^
C502	1:50	50	20	398.97 ± 48.08 ^b^
C503	1:50	50	25	474.62 ± 21.69 ^a^
C701	1:50	70	15	289.47 ± 51.50 ^def^
C702	1:50	70	20	323.15 ± 25.86 ^cde^
C703	1:50	70	25	365.62 ± 19.22 ^bc^
B301	1:30	30	15	73.94 ± 3.12 ^nop^
B302	1:30	30	20	94.35 ± 13.67 ^lmnop^
B303	1:30	30	25	101.02 ± 10.04 ^lmnop^
B501	1:30	50	15	174.38 ± 28.87 ^hij^
B502	1:30	50	20	171.39 ± 4.95 ^ijk^
B503	1:30	50	25	263.77 ± 4.40 ^efg^
B701	1:30	70	15	77.03 ± 7.22 ^mnop^
B702	1:30	70	20	89.97 ± 12.10 ^mnop^
B703	1:30	70	25	312.34 ± 18.98 ^cde^
A301	1:10	30	15	53.98 ± 1.65 ^op^
A302	1:10	30	20	76.97 ± 16.88 ^mnop^
A303	1:10	30	25	77.57 ± 2.96 ^mnop^
A501	1:10	50	15	42.73 ± 3.95 ^p^
A502	1:10	50	20	109.88 ± 7.76 ^klmno^
A503	1:10	50	25	206.31 ± 7.98 ^ghi^
A701	1:10	70	15	128.60 ± 9.55 ^jklmn^
A702	1:10	70	20	138.11 ± 16.68 ^jklm^
A703	1:10	70	25	153.29 1.81 ^ijkl^

* Extact Code. Values are expressed as mean ± standard deviation (*n* = 3). Different superscript letters indicate significant differences among treatments according to Tukey’s test at the 5% significance level. DM: dry matter.

**Table 4:** Antioxidant capacity.

	FRAP (μM Ferrous Sulfate/g)	ABTS (μM Trolox/g)
C503 *	7085.0±72.0	4921.0 ± 149.0

* Concentration: 1:50 (*w*/*v*); temperature: 50 °C; time: 25 min.

**Table 5:** pH, °Brix, and moisture during 9 days of strawberry storage.

Days	Control	GF	AF
°Brix			
1	7.5 ± 0.2 ^bD^	7.5 ± 0.1 ^bD^	9.0 ± 0.2 ^aC^
2	11.0 ± 0.2 ^aB^	11.0 ± 0.1 ^aC^	9.0 ± 0.1 ^bC^
5	12.5 ± 0.2 ^aC^	11.0 ± 0.2 ^bC^	9.0 ± 0.3 ^cC^
6	11.0 ± 0.3 ^bB^	12.0 ± 0.2 ^aB^	12.5 ± 0.2 ^aB^
7	19.0 ± 0.3 ^aA^	16.0 ± 0.3 ^bA^	12.5 ± 0.1 ^cB^
8	-	16.0 ± 0.1 ^bA^	18.5 ± 0.3 ^aA^
9	-	16.0 ± 0.1 ^A^	-
Moisture (%)			
1	92.63 ^aA^	91.39 ^aA^	89.45 ^aA^
2	90.60 ^cB^	91.46 ^bA^	92.26 ^aA^
5	88.72 ^bC^	89.04 ^bB^	89.87 ^aAB^
6	88.57 ^aBC^	82.44 ^bD^	87.97 ^aAB^
7	84.64 ^bD^	85.11 ^bC^	88.24 ^aB^
8	76.22 ^aE^	82.46 ^bC^	81.24 ^bB^
9	51.63 ^cF^	82.19 ^aD^	78.63 ^bC^

Values with different uppercase letters (vertically) differ significantly at a 5% significance level (*p* < 0.05); values with different lowercase letters (horizontally) differ significantly at 5% significance level (*p* < 0.05), with *n*= 3. GF: Gelatin-based coating; AF: starch-based coating.

## Data Availability

The original contributions presented in this study are included in the article. Further inquiries can be directed to the corresponding author.

## References

[1] Hemalatha P., Abda E.M., Shah S., Venkatesa Prabhu S., Jayakumar M., Karmegam N., Kim W., Govarthanan M. (2023). Multi-Faceted CRISPR-Cas9 Strategy to Reduce Plant Based Food Loss and Waste for Sustainable Bio-Economy—A Review. J. Environ. Manag..

[2] Pravallika K., Chakraborty S. (2022). Effect of Nonthermal Technologies on the Shelf Life of Fruits and Their Products: A Review on the Recent Trends: Shelf Life of Nonthermally Treated Fruit Products. Appl. Food Res..

[3] Taechutrakul S., Piroonpan T., Pasanphan W. (2024). Active Film Strips to Extend the Shelf Life of Fruits: Multibranched PLA-Gallic Acid as an Antioxidant/Oxygen Scavenger in a Case Study of Bananas (*Musa AAA Group*). J. Food Eng..

[4] Bajaj K., Adhikary T., Gill P.P.S., Kumar A. (2023). Edible Coatings Enriched with Plant-Based Extracts Preserve Postharvest Quality of Fruits: A Review. Prog. Org. Coat..

[5] Riva S.C., Opara U.O., Fawole O.A. (2020). Recent Developments on Postharvest Application of Edible Coatings on Stone Fruit: A Review. Sci. Hortic..

[6] Fakhouri F.M., Martelli S.M., Caon T., Velasco J.I., Mei L.H.I. (2015). Edible Films and Coatings Based on Starch/Gelatin: Film Properties and Effect of Coatings on Quality of Refrigerated Red Crimson Grapes. Postharvest Biol. Technol..

[7] Nogueira G.F., Fakhouri F.M., de Oliveira R.A. (2019). Effect of Incorporation of Blackberry Particles on the Physicochemical Properties of Edible Films of Arrowroot Starch. Dry. Technol..

[8] Fakhouri F.M., Nogueira G.F., de Oliveira R.A., Velasco J.I. (2019). Bioactive Edible Films Based on Arrowroot Starch Incorporated with Cranberry Powder: Microstructure, Thermal Properties, Ascorbic Acid Content and Sensory Analysis. Polymers.

[9] Beikzadeh S., Khezerlou A., Jafari S.M., Pilevar Z., Mortazavian A.M. (2020). Seed Mucilages as the Functional Ingredients for Biodegradable Films and Edible Coatings in the Food Industry. Adv. Colloid Interface Sci..

[10] da Silva J.B.A., Pereira F.V., Druzian J.I. (2012). Cassava Starch-Based Films Plasticized with Sucrose and Inverted Sugar and Reinforced with Cellulose Nanocrystals. J. Food Sci..

[11] Yacob N., Yusof M.R., Mohamed A.Z., Badri K.H. (2019). Effect of Cellulose Fiber Loading on the Properties of Starch-Based Films. AIP Conf. Proc..

[12] Fakhouri F.M., Costa D., Yamashita F., Martelli S.M., Jesus R.C., Alganer K., Collares-Queiroz F.P., Innocentini-Mei L.H. (2013). Comparative Study of Processing Methods for Starch/Gelatin Films. Carbohydr. Polym..

[13] Dou L., Li B., Zhang K., Chu X., Hou H. (2018). Physical Properties and Antioxidant Activity of Gelatin-Sodium Alginate Edible Films with Tea Polyphenols. Int. J. Biol. Macromol..

[14] Mohammed H.A., Eddine L.S., Souhaila M., Hasan G.G., Kir I., Abdullah J.A.A. (2024). Green Synthesis of SnO_2_ Nanoparticles from *Laurus Nobilis L*. Extract for Enhanced Gelatin-Based Films and CEF@SnO_2_ for Efficient Antibacterial Activity. Food Bioprocess Technol..

[15] Andreuccetti C., Carvalho R.A., Galicia-García T., Martinez-Bustos F., González-Nuñez R., Grosso C.R.F. (2012). Functional Properties of Gelatin-Based Films Containing *Yucca schidigera* Extract Produced via Casting, Extrusion and Blown Extrusion Processes: A Preliminary Study. J. Food Eng..

[16] Johnston-Banks F.A. (1990). Gelatine. Food Gels.

[17] Podshivalov A., Zakharova M., Glazacheva E., Uspenskaya M. (2017). Gelatin/Potato Starch Edible Biocomposite Films: Correlation between Morphology and Physical Properties. Carbohydr. Polym..

[18] de Lima R.P., Carrea D.d.A., Garcia V.A.d.S., Tostes Filgueiras C., Matta Fakhouri F., Velasco J.I. (2025). Development of Gelatin-Based Renewable Packaging with *Melaleuca alternifolia* Essential Oil for Chicken Breast Preservation. Polymers.

[19] Matta Fakhouri F., Batista Alves J., Grosso Ferreira C.R. (2003). Efeito de Coberturas Comestíveis Aplicadas Em Goiabas in Natura (*Psidium guajava*). I. Desenvolvimento e Caracterização de Filmes Comestíveis de Gelatina, Triacetina e Ácidos Graxos. Braz. J. Food Technol..

[20] Trabelsi A., El Kaibi M.A., Abbassi A., Horchani A., Chekir-Ghedira L., Ghedira K. (2020). Phytochemical Study and Antibacterial and Antibiotic Modulation Activity of *Punica granatum* (Pomegranate) Leaves. Scientifica.

[21] Singh J., Kaur H.P., Verma A., Chahal A.S., Jajoria K., Rasane P., Kaur S., Kaur J., Gunjal M., Ercisli S. (2023). Pomegranate Peel Phytochemistry, Pharmacological Properties, Methods of Extraction, and Its Application: A Comprehensive Review. ACS Omega.

[22] Machado J.C.B., Cristina da Silva J., Leite G.V.B., dos Santos Dantas T., Daniele-Silva A., de Freitas Fernandes-Pedrosa M., de Oliveira A.M., Dantas da Cruz R.C., de Souza I.A., Weilack I. (2025). Phytochemical Profile, Acute, and Subacute Toxicity of Spray-Dried Hydroethanolic Extract From *Punica granatum* Leaves. Chem. Biodivers..

[23] Ahmad F., Kumar A., Faizan A., Amardeep K. (2018). Optimization of the Ultrasonic Assisted Extraction Process to Obtain Phenolic Compounds from Pomegranate (*Punica granatum*) Peels Using Response Surface Methodology. Int. J. Agric. Sci..

[24] Foujdar R., Bera M.B., Chopra H.K. (2020). Optimization of Process Variables of Probe Ultrasonic-assisted Extraction of Phenolic Compounds from the Peel of *Punica granatum* Var. Bhagwa and It’s Chemical and Bioactivity Characterization. J. Food Process. Preserv..

[25] Živković J., Šavikin K., Janković T., Ćujić N., Menković N. (2018). Optimization of Ultrasound-Assisted Extraction of Polyphenolic Compounds from Pomegranate Peel Using Response Surface Methodology. Sep. Purif. Technol..

[26] Lazzarotto R.S.S., Stasyszen De Freitas Scherruth M., Calixto P.S., Carraro M.M.D., Silveira A.C.D., Lazzarotto M. (2020). Folin Ciocalteau Adapted Method to Quantify Polyphenols in Yerba Mate Extracts.

[27] Rufino M.d.S.M., Alves R.E., Brito E.S., Morais S.M., Sampaio C.d.G., Perez-Jimenez J., Saura-Calixto F.D. (2006). Metodologia Científica: Determinação da Atividade Antioxidante Total em Frutas pelo Método de Redução do Ferro (FRAP).

[28] Rufino M.d.S.M., Alves R.E., Sousa De Brito E., Maia De Morais S., De Goes Sampaio C., Pérez-Jiménez J., Saura-Calixto F.D. (2007). Determinação da Atividade Antioxidante Total em Frutas pela Captura do Radical Livre ABTS^+^.

[29] (2012). CLSI Performance Standards for Antimicrobial Disk Susceptibility Tests. Approved—Eleventh Edition.

[30] (2004). CLSI Method for Antifungal Disk Diffusion Susceptibility Testing of Yeasts; Approved Guideline.

[31] (2010). CLSI Method for Antifungal Disk Diffusion Susceptibility Testing of Nondermatophyte Filamentous. Fungi; Approved Guideline.

[32] (2005). AOAC Official Method of Analysis.

[33] Balamurugan C., Karuppasamy R., Sivaraj C., Saraswathi K., Arumugam P. (2020). *Punica granatum* L. (Pomegranate) Leaves Extract: The Study of Antioxidant and Antibacterial Activity. J. Pharmacogn. Phytochem..

[34] Sharayei P., Azarpazhooh E., Zomorodi S., Ramaswamy H.S. (2019). Ultrasound Assisted Extraction of Bioactive Compounds from Pomegranate (*Punica granatum* L.) Peel. LWT.

[35] Morzelle M.C. (2012). Resíduos de Romã (Punica granatum) na Prevenção da Doença de Alzheimer.

[36] Garima P., Akoh C.C. (2009). Antioxidant Capacity and Lipid Characterization of Six Georgia-Grown Pomegranate Cultivars. J. Agric. Food Chem..

[37] Nag S., Sit N. (2018). Optimization of Ultrasound Assisted Enzymatic Extraction of Polyphenols from Pomegranate Peels Based on Phytochemical Content and Antioxidant Property. J. Food Meas. Charact..

[38] Yu M., Gouvinhas I., Barros A. (2021). Variation of the Polyphenolic Composition and Antioxidant Capacity of Freshly Prepared Pomegranate Leaf Infusions over One-Day Storage. Antioxidants.

[39] Pengkumsri N., Kaewdoo K., Leeprechan W., Sundaram Sivamaruthi B. (2019). Influence of Extraction Methods on Total Phenolic Content and Antioxidant Properties of Some of the Commonly Used Plants in Thailand. Pak. J. Biol. Sci..

[40] Silva V., Silva A., Ribeiro J., Aires A., Carvalho R., Amaral J.S., Barros L., Igrejas G., Poeta P. (2023). Screening of Chemical Composition, Antimicrobial and Antioxidant Activities in Pomegranate, Quince, and Persimmon Leaf, Peel, and Seed: Valorization of Autumn Fruits By-Products for a One Health Perspective. Antibiotics.

[41] Nascimento K., Batista Da Silva E., Barbosa M.J. (2013). Determination of Antioxidant Activity of Pomegranate (*Punica granatum* L.) by Methods DPPH and FRAP. Hig. Aliment..

[42] Machado J.C.B., Ferreira M.R.A., Soares L.A.L. (2023). *Punica granatum* Leaves as a Source of Active Compounds: A Review of Biological Activities, Bioactive Compounds, Food, and Technological Application. Food Biosci..

[43] Zebua D.N., Prima E.C., Yelliantty, Garnida Y. (2025). Effect of a Pectin Edible Coating with Lemon Peel Extract to Maintain Strawberry Fruit’s Quality during Cold Storage. Food Humanit..

[44] Françoso L.L.T., Couto M.A.L., Canniatti-Brazaca S.G., Arthur V. (2008). Physical-Chemical Alterations in Irradiated and Stored Strawberries (*Fragaria Anassa Duch.*). Food Sci. Technol..

[45] da Silva M.C.R., Schmidt V.C.R. (2015). Avaliação da vida-de-prateleira de morangos recobertos com biofilme de acetato de amido e acetato de amido com adição de sorbato de potássio. Proceedings of the Anais do Congresso Brasileiro de Engenharia Química em Iniciação Científica—Cobeq IC 2015.

[46] Korte K.P., Favarão S.C.M. (2016). Effect of Gelatine Colourless and Commercial Associated with Plant Extracts As Edible Coating in Strawberry Post-Harves. Rev. Campo Digit..

[47] Aitboulahsen M., Zantar S., Laglaoui A., Chairi H., Arakrak A., Bakkali M., Zerrouk M.H. (2018). Gelatin-Based Edible Coating Combined with *Mentha Pulegium* Essential Oil as Bioactive Packaging for Strawberries. J. Food Qual..

